# An Agreement of Antigen Tests on Oral Pharyngeal Swabs or Less Invasive Testing With Reverse Transcription Polymerase Chain Reaction for Detecting SARS-CoV-2 in Adults: Protocol for a Prospective Nationwide Observational Study

**DOI:** 10.2196/35706

**Published:** 2022-05-04

**Authors:** Uffe Vest Schneider, Jenny Dahl Knudsen, Anders Koch, Nikolai Søren Kirkby, Jan Gorm Lisby

**Affiliations:** 1 Department of Clinical Microbiology Copenhagen University Hospital Hvidovre Hvidovre Denmark; 2 Department of Clinical Microbiology Rigshospitalet Copenhagen Denmark; 3 Department of Infectious Diseases Rigshospitalet Copenhagen Denmark

**Keywords:** SARS-CoV-2, COVID-19, point of care, PoC, antigen test, anatomic sampling location, Reverse Transcription Polymerase Chain Reaction, RT-PCR, rapid antigen test, RAT, testing, antigen, sampling, PCR, rapid, protocol, prospective, observational, agreement, oral, adult, sensitivity, specificity, test location, anatomy, saliva, swab, nasopharyngeal, nasal

## Abstract

**Background:**

The SARS-CoV-2 pandemic has resulted in an unprecedented level of worldwide testing for epidemiologic and diagnostic purposes, and due to the extreme need for tests, the gold-standard Reverse Transcription Polymerase Chain Reaction (RT-PCR) testing capacity has been unable to meet the overall worldwide testing demand. Consequently, although the current literature has shown the sensitivity of rapid antigen tests (RATs) to be inferior to RT-PCR, RATs have been implemented on a large scale without solid data on performance.

**Objective:**

This study will compare analytical and clinical sensitivities and specificities of 50 lateral flow– or laboratory-based RATs and 3 strand invasion–based amplification (SIBA)-RT-PCR tests from 30 manufacturers to RT-PCR testing of samples obtained from the deep oropharynx. In addition, the study will compare sensitivities and specificities of the included RATs as well as RT-PCR on clinical samples obtained from the deep oropharynx, the anterior nasal cavity, saliva, the deep nasopharynx, and expired air to RT-PCR on deep oropharyngeal samples.

**Methods:**

In the prospective part of the study, 200 individuals found SARS-CoV-2 positive and 200 individuals found SARS-CoV-2 negative by routine RT-PCR testing will be retested with each RAT, applying RT-PCR as the reference method. In the retrospective part of the study, 304 deep oropharyngeal cavity swabs divided into 4 groups based on RT-PCR quantification cycle (Cq) levels will be tested with each RAT.

**Results:**

The results will be reported in several papers with different aims. The first paper will report retrospective (analytical sensitivity, overall and stratified into different Cq range groups) and prospective (clinical sensitivity) data for RATs, with RT-PCR as the reference method. The second paper will report results for RAT based on anatomical sampling location. The third paper will compare different anatomical sampling locations by RT-PCR testing. The fourth paper will focus on RATs that rely on central laboratory testing. Tests from 4 different manufacturers will be compared for analytical performance data on retrospective deep oropharyngeal swab samples. The fifth paper will report the results of 4 RATs applied both as professional use and as self-tests. The last paper will report the results from 2 breath tests in the study. A comparison of sensitivity and specificity between RATs will be conducted using the McNemar test for paired samples and the chi-squared test for unpaired samples. Comparison of the positive predictive value (PPV) and negative predictive value (NPV) between RATs will be performed by the bootstrap test, and 95% CIs for sensitivity, specificity, PPV, and NPV will be calculated as bootstrap CIs.

**Conclusions:**

The study will compare the sensitivities of a large number of RATs for SARS-CoV-2 to with those of RT-PCR and will address whether lateral flow–based RATs differ significantly from laboratory-based RATs. The anatomical test locations for both RATs and RT-PCR will also be compared.

**Trial Registration:**

ClinicalTrials.gov NCT04913116; https://clinicaltrials.gov/ct2/show/NCT04913116

**International Registered Report Identifier (IRRID):**

DERR1-10.2196/35706

## Introduction

### Background

The SARS-CoV-2 pandemic has resulted in an unprecedented level of worldwide testing for epidemiologic and diagnostic purposes. In Denmark, at the peak of testing activity in week 20 in 2021, a total of 615,000 individuals (10.6% of the total population of 5.8 million) were tested daily by Reverse Transcription Polymerase Chain Reaction (RT-PCR) or rapid antigen tests (RATs). PCR and PCR-like technologies (nucleic acid amplification technologies [NAATs]) are considered the gold standard for detection of viral pathogens, but due to the extreme need for tests, the national RT-PCR testing capacity was unable to meet the overall testing demand. Consequently, RATs, which may be performed by non-health-care-trained individuals outside of health care facilities with results within minutes, were implemented on a large scale. At the peak testing level, 440,000 RATs were performed daily in Denmark compared to 175,000 RT-PCR tests. This may be problematic, however, as unlike RT-PCR tests, which are clinically validated, the only information available about the sensitivity of a specific RAT is most often the manufacturer’s instructions for use (IFU).

### Previous Experience With RATs for Infectious Diseases

Prior to the SARS-CoV-2 pandemic, commercially available RATs were primarily based on the lateral flow immunoassay principle first described by Leuvering et al [1] in 1980 and are available for a number of pathogens [2]; especially point-of-care (PoC) assays for detection of malaria [3,4], group A streptococcus [5], respiratory syncytial virus [6,7] and influenza virus types A and B [7,8] have been widely implemented. In a meta-analysis describing the performance of rapid diagnostic tests for group A streptococcal pharyngitis [5], lanthanide immunofluorescent assay (LIFA) and optical immunoassay (OIA) were performing similarly when individual studies were pooled with an average sensitivity of 85% and an average specificity of 97% against culture, but the assays performed with large differences between different suppliers. The sensitivity varied from 71% to 95%, and the specificity varied from 62% to 100% [9-11]. In a meta-analysis published by Chartrand et al [8] describing the performance of rapid diagnostic tests for influenza virus, in 67 studies where RT-PCR was the comparator, a pooled sensitivity of 54% and a pooled specificity of 99% were reported. A significantly higher sensitivity was reported in studies showing results from children compared to studies reporting results from adults. In a meta-analysis published by Merckx et al [7], 94 studies on influenza A and 30 studies on influenza B applying traditional lateral-flow technology compared to RT-PCR showed a pooled sensitivity of 54.4% for influenza A and 53.2% for influenza B and a pooled specificity of 99.4% for influenza A and 99.8% for influenza B. When newer digital immunoassays were applied (18 studies on influenza A and 17 studies on influenza B), a pooled sensitivity of 80.0% for influenza A and 76.8% for influenza B and a pooled specificity of 98.3% for influenza A and 98.7% for influenza B were reported. Again, assays performed on children showed a significantly higher sensitivity compared to assays performed on adults. In a meta-analysis published by Bruning et al [6], 134 studies on influenza and 32 studies on respiratory syncytial virus applying rapid immunoassays compared to RT-PCR showed a pooled sensitivity of 61.1% and a pooled specificity of 98.8% for influenza. For respiratory syncytial virus, the pooled sensitivity was higher (75.3%) and the pooled specificity was 98.7% [6]. The authors reported huge differences between different commercial influenza assays (ie, 36% for Directigen Flu A+B [BD Diagnostic Systems] and 44% for QuickVue Influenza A+B [Quidel] and BinaxNow Influenza A&B [Alere] to 75% for Sofia Influenza A+B [Quidel] and 76% for mariPOC [ArcDia International]). However, for respiratory syncytial virus, the difference in sensitivity between the reported assays was not significant.

### Background on RATs for SARS-CoV-2

Thus, prior to the SARS-CoV-2 pandemic, all available PoC immunoassays for other pathogens have shown inferior performance compared to NAATs [5-8]. Immunoassays in general show excellent specificity but inferior sensitivity and should, for diagnostic purposes, be used as rule-in assays only [12,13]. Early in the SARS-CoV-2 pandemic, RATs based on lateral-flow technology were made commercially available, and new commercial RATs for detection of SARS-CoV-2 are continuously being introduced. The literature on analytical as well as clinical sensitivity is scarce; most often, a single RAT is compared against a NAAT. The sensitivity of individual RATs varies according to population (eg, symptomatic vs nonsymptomatic) and timing related to symptoms (if any) [14] and may be as low as 37.7%. Interestingly, according to the manufacturers’ own reported data, 125 commercial tests all had a clinical sensitivity of >80.6% (range 80.6%-100%, average 95.69%, median 96.17%) [15]. Of the 125 tests, 121 (99.2%) had a sensitivity of >90% according to the manufacturers’ own reported data.

Considering the extreme number of worldwide performed RATs, the lack of thorough comparisons of the performances of different RATs is problematic, as decision makers have so far spent large amounts of financial resources based on limited objective and validated data.

### Research Aims

In this study, we will compare the analytical as well as the clinical sensitivities and specificities of 50 RATs and 3 strand invasion–based amplification (SIBA)-RT-PCR tests from 30 manufacturers to RT-PCR on deep oropharyngeal samples, which is the chosen anatomical sampling site for routine SARS-CoV-2 RT-PCR in Denmark. In addition, we will compare the clinical sensitivities and specificities of all included RATs as well as RT-PCR on clinical samples obtained from the deep oropharyngeal cavity, the anterior nasal cavity, saliva, the deep nasopharyngeal cavity, and expired air to RT-PCR on deep oropharyngeal samples.

## Methods

### Summary of Design

This research combines a retrospective study of analytical sensitivity and specificity with a prospective accuracy observational study. In the prospective part of the study, approximately 200 individuals testing positive for SARS-CoV-2 and 200 individuals testing negative for SARS-CoV-2 by routine SARS-CoV-2 testing will be subsequently retested with each RAT, with RT-PCR on deep oropharyngeal cavity swabs as the reference method.

In the retrospective part of the study, 304 deep oropharyngeal cavity swabs stored in Universal Transport Medium (UTM; Copan Diagnostics Inc, Brescia, Italy) will be divided into 4 groups based on RT-PCR quantification cycle (C_q_) levels and tested with each included RAT.

### Recruitment

Individuals who test positive for SARS-CoV-2 by a public test provider, either TestCenter Denmark (TCDK; national complimentary screening for SARS-CoV-2) or a regional department of clinical microbiology (DCM; health care workers, residents at nursing homes, outpatients, individuals undergoing elective surgery, and hospitalized patients) will be identified directly in the local microbiology laboratory information systems. SARS-CoV-2-positive individuals will be invited to participate in the study either by phone or by email. Twice daily (approximately at 8:30 AM and 8:30 PM), all individuals who have tested positive within the past 12 h and live in a geographic area in which the project includes patients will be invited by secure public email (e-Boks) to participate in the study and asked to contact a test coordinator by email or phone within 24 h. Each SARS-CoV-2-positive individual will be asked to provide information about the test date, current address, and contact information to allow the test coordinator to plan the new testing in the individual’s private home by an outpatient testing team. All individuals will be retested within 72 h after the first initial positive test for SARS-CoV-2.

### Data Management

Written consent for participation and collection of data in the study will be collected at the test center. For consecutive samples, each RAT and the sample for RT-PCR will be marked with a local sample identifier number and data will be paired according to this sample identification number. Individuals who test positive for SARS-CoV-2 will be invited by email or phone to participate in additional testing. The number of individuals invited to participate will be registered, without any additional information. For additional testing, a new sample identifier number will be used to pair the new swab(s) for RT-PCR testing and RATs conducted by the outpatient team. Data, including photodocumentation of all RAT results, will be sent to the steering group and stored in a secure regional electronic file archive.

When retested by the outpatient testing team, a signed consent form for participation will be collected and no other information about the participating individual will be stored afterward. SARS-CoV-2-positive and SARS-CoV-2-negative individuals will be included until approximately 200 RT-PCR-positive and RT-PCR-negative individuals have been included for each RAT.

### Data Validation

The results of all RATs will be documented by picture, and all results will subsequently be reviewed by the test coordinator. The test coordinator will inspect all pictures and change negative results to positive if a visual target band is present on the pictures of the RAT. If the target band cannot be verified, the original interpretation by the outpatient testing team will be sustained, as the outpatient testing team will get the opportunity to see the test from multiple angels and thereby to detect the sample band on the RAT, whereas the test coordinator is limited by review of the picture documentation. For RT-PCR results, all samples negative for SARS-CoV-2 and a human control gene will be considered inconclusive and curves will be inspected by RT-PCR staff a second time to ensure that results are reported correctly.

### Sampling and RATs

Participating individuals will be tested by RATs as well as RT-PCR on clinical samples obtained from the anterior nasal cavity, the deep oropharyngeal cavity, saliva, the deep nasopharyngeal cavity, and expired air. Sampling for RATs will be performed using the sterile swabs provided by each manufacturer and will be executed according to the instructions from the manufacturer. If no special instructions are provided and for RT-PCR, samples will be collected as specified next. By default, RATs will be performed on deep oropharyngeal cavity swabs, as deep nasopharyngeal cavity swabbing is not recommended in Denmark due to the discomfort for the individual being tested. In addition to deep oropharyngeal cavity testing, manufacturers could submit their RATs for testing on other sample material, such as swabs from the anterior nasal cavity, saliva, or expired air.

Each RAT will be executed as instructed by the manufacturer in the IFU. When non-CE marked sample materials are used, sampling will be conducted as instructed by the manufacturer using swabs and sampling media delivered specifically by the manufacturer. All RATs will be performed on-site immediately after sample collection. The results will be collected on a test chart, and a photo will be taken of the RAT for documentation of results. Self-tests will be performed by the tested individuals themselves 2 h following the sampling procedure performed by the outpatient sampling team. For self-testing, tested individuals will be asked to send a picture of the test strip, together with their interpretation of the test result to the test coordinator.

#### Anterior Nasal Cavity Swabs

Up to 3 sterile swabs will be inserted into the anterior nasal cavity at a time. The swabs will be inserted 2-3 cm from the nostril, aiming below the inferior turbinate, and rotated 5 times in each nostril to collect sample material. Swabs for RATs will be tested immediately after sample collection, whereas sterile flocked nylon swabs for RT-PCR will be transferred to a NEST disposable sampler inactivation transport medium (NEST tube and Scientific Nasopharyngeal Specimen Collection Swab, Wuxi NEST Biotechnology Co., Ltd, Wuxi City, Jiangsu Province, China) and sent for RT-PCR.

#### Deep Nasopharyngeal Cavity Swabs

Samples for deep nasopharyngeal cavity testing will be collected only for RT-PCR to compare anatomical testing locations. A sterile flocked nylon swab (NEST Scientific Nasopharyngeal Specimen Collection Swab, cat. no. 202004) with 1 break point at 8 cm will be inserted below the inferior turbinate until it reaches the posterior nasopharyngeal wall. The swab will be rotated 5 times, and the collected sample material will be transferred to a NEST tube and sent for RT-PCR.

#### Deep Oropharyngeal Cavity Swabs

Testing will be conducted with 3 oropharyngeal swabs at a time in a tree point swab procedure. The 3 swabs will be held together and rotated at both sides of the palatoglossal arch and the posterior wall of the oropharynx. The tongue or teeth will be avoided, and all 3 areas will need to be sampled. Of the 3 swabs, 2 will be tested immediately by RATs, whereas the third flocked nylon swab will be sent for RT-PCR in a NEST tube (Oropharyngeal Specimen Collection Swab, cat. no. 202003, Wuxi NEST Biotechnology Co, Ltd).

#### Saliva Sample Collection

Saliva samples will be collected according to the manufacturer’s instructions. If no specific instruction is provided or for RT-PCR, the sample will be collected by instructing the person being tested to massage the glands on both sides of the jaw and them sampling the saliva produced from the parotid, submandibular, and sublingual glands. Tested persons will be instructed to place the tongue on the hard palate and bow the head forward to let saliva be secreted naturally into a plastic cup/saliva collector tube while massaging. Saliva for RT-PCR will be collected in a NEST Scientific Saliva Collection Kit with inactivating transport media (cat. no. 203011).

#### Expired Air Collection

Collection of expired air will be performed according to the manufacturer’s instructions and will be described in detail when the results are reported. Results will be recorded from 2 different tests on expired air.

### RT-PCR Testing at the Danish Technology University

Participants in the prospective part of the study will be either retested after invitation or tested as part of surveillance/diagnostics for SARS-CoV-2. A sample from the latter group will be locally tested at the local DCM or sent to the TCDK for RT-PCR. For all participants, an oropharyngeal sample in a NEST tube will be sent to the test center at Technical University of Denmark (DTU), Lyngby, to ensure that all participating individuals are tested similarly, and all RATs will be compared to the same reference RT-PCR. To evaluate the anatomical testing site for RT-PCR, additional samples will be obtained from the deep nasopharyngeal cavity, the anterior nasal cavity, the deep oropharyngeal cavity, and saliva in NEST tubes and sent to the DTU for RT-PCR testing.

At the DTU test center, all samples will be tested for SARS-CoV-2 by applying the CoviDetect – COVID-19 Multiplex RT-PCR assay from PentaBase (PentaBase APS, Odense, Denmark). In short, samples will be received in NEST tubes (3 M guanidine thiocyanate buffer for instant viral lysis). Viral RNA will be purified from a 200 µL sample on a Beckman Coulter Biomek i7 (Beckman Coulter Life Sciences, Indianapolis, NV, USA) with magnetic bead–based purification (RNAadvance Viral XP kit, Beckman Coulter, Indianapolis, NV, USA) with a 30 µL eluate. Next, 5 µL of the purified RNA sample will be mixed with 10 µL of 2x Mastermix One Step PrimeScript III, RT-PCR mix (cat. no. RR600; TaKaRa Bio Europe AB, Göteborg, Sweden) and 5 µL of the 4x primer-probe mix. The assay includes 2 targets in the nucleocapsid protein gene (N-gene) of SARS-CoV-2 and 1 target for the human RNase P gene (RP-gene) as a process control and to confirm the presence of human DNA in the sample (Table 1).

Samples will be amplified by a 2-step touch-down RT-PCR program on a Rotor_Gene (Qiagen Aarhus, Aarhus, Denmark) with reverse transcription at 52°C for 5 min, initial PCR activation at 95°C for 10 s, 7 cycles of denaturation at 95°C for 5 s with annealing/elongation at 66°C for 30 s, and finally 38 cycles of denaturation at 95°C for 5 s and 60°C for 30 s.

**Table 1:** Primer and probe sequences for the 2 N-gene^a^ targets included in the CoviDetect multiplex assay. Sequences for the human RP-gene^b^ (marked with Cy5^c^) or concentrations are not reported by the manufacture.

Oligonucleotide name	Sequence (5’-3’)
N1 forward primer	GACCCCAAAATCAGCGAAAT
N1 reverse primer	CGCAGTATTATTGGGTAAACC
N1 probe (5’-FAM^d^/3’-unknown)	ACCCCGCATTACGTTTGGTGGACC
N2 forward primer	AGGAACTGATTACAAACATTGGC
N2 reverse primer	TGTAGGTCAACCACGTTCCC
N2 probe (5’-HEX^e^/3’-unknown)	TGCACAATTTGCCCCAGCG

^a^RP-gene: RNase P gene.

^b^N-gene: nucleocapsid protein gene.

^c^Cy5: cyanine fluorophore.

^d^FAM: 6-carboxyfluorescein fluorophore.

^e^HEX: hexachloro-fluorescein fluorophore.

### RT-PCR at the TCDK

To evaluate whether the anatomical test location is independent of the RT-PCR of choice and test media, all samples for comparison of anatomical locations will be sent to the TCDK for repeated RT-PCR testing. Additional samples will be collected from the deep oropharyngeal cavity, the anterior nasal cavity, and saliva. Deep oropharyngeal cavity and anterior nasal cavity swabbing will be conducted as described before. Anterior nasal cavity swabbing will be either performed as professional testing or self-administered testing under supervision. The salivary swab will be collected by placing the swab on the volunteer’s tongue for 10 s without any stimulation of the salivary glands. The same type of flocked swab will be used for sample collecting from all 3 anatomical testing locations (CLASSIQSwab Sterile Dry Fiber Swabs, Copan Diagnostics Inc), and all samples will be collected in individual screw cap–sealed 1000 µL 2D barcoded tubes (LVL Technologies, Crailsheim, Germany) without stabilizing or transport media. Samples will be delivered and analyzed within 24 h of sample collection at the TCDK.

The sample material will be suspended from the swabs directly in sample tubes using a Hamilton Microlab VANTAGE (Hamilton Company, Reno, NV, USA) liquid-handling system by trained laboratory technicians. Individual swabs will be suspended in 700 µL of 1X Dulbecco’s phosphate-buffered saline (DPBS; Gibco, Thermo Fischer Scientific, Waltham, MA, USA) and agitated at 700 RPM for 10 min, and 200 µL will be transferred to deep-well plates for downstream processing.

RNA extraction and purification will be carried out using a Beckman Coulter RNAdvance Blood kit on a Beckman Coulter Biomek i7 automated workstation with 200 µL sample input and 50 µL elution volume. From this, 5 µL eluate will be transferred to a 96-well skirted PCR plate.

Each PCR tube will contain 12.5 µL of Luna Universal Probe One-Step RT-PCR Kit reaction buffer, 1.25 µL of Luna WarmStart RT Enzyme mix (New England Biolabs Inc, Ipswich, MA, USA) primers and probes targeting the envelope gene (E-gene) [16] (at 100 µM, volumes and sequences in Table 2), 5 µL of the template, and DNAse/RNAse-free water for a total volume of 25 µL.

Single-target RT-PCR assays will be performed on a Bio-Rad CFX96 Touch Real-Time PCR Detection System (Bio-Rad Laboratories, Hercules, CA, USA) using the software CFX Maestro (Bio-Rad Laboratories). The cycling conditions will be reverse transcription at 55°C for 10 min and initial denaturation at 95°C for 3 min, followed by 45 cycles of denaturation and annealing/extension at 95°C for 15 s and 58°C at 30 s, respectively.

**Table 2:** Primers and probe sequences and final concentrations of the oligonucleotides targeting the E-gene^a^ by TCDK^b^ RT-PCR^c^.

Oligonucleotide name	Sequence (5’-3’)	Final concentration (nM)
E_Sarbeco_F	ACAGGTACGTTAATAGTTAATAGCGT	400
E_Sarbeco_R	ATATTGCAGCAGTACGCACACA	400
E_Sarbeco_P1	FAM^d^-ACACTAGCCATCCTTACTGCGCTTCG-BHQ1^e^	200

^a^E-gene: envelope gene.

^b^TCDK: TestCenter Denmark.

^c^RT-PCR: Reverse Transcription Polymerase Chain Reaction.

^d^FAM: 6-carboxyfluorescein fluorophore.

^e^BHQ1: Black Hole Quencher 1.

### Retrospective Testing

Samples will be stored in UTM to ensure that they can be used for subsequent antigen testing as well as subsequent retesting by RT-PCR. We will use laboratory-developed test (LDT) RT-PCR that reports threshold cycle (C_t_) values in order to be able to stratify the samples into different C_q_ range groups directly from aliquoted samples that will also be used for RATs.

For the retrospective study arm, stored excess material from 204 previously SARS-CoV-2-positive and 100 SARS-CoV-2-negative samples stored at –80°C will be defrosted on ice and retested by RT-PCR to verify the C_q_ value. Selected samples will be defrosted and adjusted with UTM (at 4°C; Copan Diagnostics Inc) or pooled as multiple negative samples, again on ice, and aliquots of 250-500 µL volume will be stored at –80°C until use.

Negative samples will be prepared from routine samples obtained from 10 individuals who test negative in routine RT-PCR. The samples will be pooled and aliquoted into 40 vials of 500 µL. Positive samples will be adjusted to a certain C_q_ range as either 1 part sample plus 3 parts UTM, which will be conducted for 186 samples, or as 1 part sample plus 7 parts UTM, which will be conducted for 13 samples. Of these 13 samples, 1 (8%) has a final C_q_ between 30 and 35, and the remaining 12 (92%) have a C_q_ of >35. In addition, 5 samples will be diluted as 1 part sample plus 11 parts UTM. Of these 5 samples, 1 (20%) sample has a C_q_ of <25 and 4 (80%) have a C_q_ between 25 and 30 each in our LDT RT-PCR. The final 204 samples will be stored as 50 (24.5%) positive samples with a C_q_ of <50, 54 (26.5%) samples with a C_q_ between 25 to 30, 50 (24.5%) samples with a C_q_ between 30 and 35, and 50 (24.5%) samples with a C_q_ level of >35.

For each RAT, 1 aliquot of each sample will be defrosted, and 200 µL of the sample material will be transferred to RAT lysis buffer and tested according to the manufacturer’s instructions by laboratory-trained personnel; 1 aliquot will be used for each RAT, and excess material will be discharged; and 1 aliquot will be retested by RT-PCR after thawing to verify the C_q_ of the sample.

In short, 180 µL of the sample material will be purified on a MGISP-960 purifier with a MGIEasy Magnetic Beads Virus DNA/RNA extraction kit (MGI Tech Co, Ltd, Shenzhen, China) and a final eluate of 33 µL purified DNA/RNA. Next, 8 µL of purified RNA will be mixed with 10 µL of 2x KiCqStart One-Step Probe RT-PCR ReadyMix from Sigma-Aldrich (Merck Life Science A/S, Søborg, Denmark), 1 µL of 2'-deoxyuridine 5'-triphosphate (dUTP; 4 mM), and 1 µL of primer-probe mix targeting the E-gene and N-gene of SARS-CoV-2 and the human RP gene as process and sampling controls (Table 3) [16,17].

RT-PCR will be performed on a LineGene 9600-platform (Hangzhou Bioer Technology Co, Ltd, Hangzhou, China) with reverse transcription at 50°C for 10 min, reverse transcription inactivation/initial denaturation at 95°C for 60 s, and 45 cycles of denaturation at 95°C for 5 s, followed by annealing/elongation at 60°C for 30 s.

**Table 3:** Primers and probe sequences and final concentrations of the oligonucleotides targeting the E-gene^a^, N-gene^b^, and human RP-gene^c^ by LDT^d^ RT-PCR^e^.

Oligonucleotide name	Sequence (5’-3’)	Final concentration (nM)
E_Sarbeco_F	ACAGGTACGTTAATAGTTAATAGCGT	500
E_Sarbeco_R	ATATTGCAGCAGTACGCACACA	400
E_Sarbeco_P	LC610^f^-ACACTAGCCATCCTTACTGCGCTTCG-BBQ^g^	150
N2_CDC_F	TTACAAACATTGGCCGCAAA	400
N2_TibMol_R1	AAGGTGTGACTTCCATGCCA	400
N2_CoV2_P	FAM^h^- ACAATTTGCCCCCAGCGCTTCAG-BBQ	150
H_RnaseP_F	AGATTTGGACCTGCGAGCG	100
H_RnaseP_R	GAGCGGCTGTCTCCACAAGT	100
H_RnaseP_P	Cy5^i^- TTCTGACCTGAAGGCTCTGCGCG-BBQ	125

^a^E-gene: envelope gene.

^b^N-gene: nucleocapsid protein gene.

^c^RP-gene: RNase P gene.

^d^LDT: laboratory-developed test.

^e^RT-PCR: Reverse Transcription Polymerase Chain Reaction.

^f^LC610: LightCycler Red 610 fluorophore.

^g^BBQ: BlackBerry Quencher.

^h^FAM: 6-carboxyfluorescein fluorophore.

^i^Cy5: cyanine fluorophore.

### Statistical Analysis

#### Prospective Study Arm

The performance of each RAT will be reported as sensitivity, specificity, positive predictive value (PPV), and negative predictive value (NPV) compared to oropharyngeal swabs evaluated by RT-PCR. Performance of RATs will further be evaluated regarding RT-PCR results and stratified into 3 different C_q_ ranges as strong positive (C_q_<15), intermediate positive (C_q_=15-20), and weak positive (C_q_>20).

Among consecutively collected prospective samples, the fraction of samples testing negative for human DNA by RT-PCR will be reported.

#### Retrospective Study Arm

Determination of analytical sensitivity and specificity will be stratified into 4 groups by RT-PCR (C_q_=20-25, C_q_=25-30, C_q_=30-35, and C_q_>35).

Comparison of sensitivity and specificity between RATs will be performed using the McNemar test for paired samples and the chi-squared test for unpaired samples. The level of significance will be 0.05. Comparison of the PPV and NPV between RATs will be performed using the bootstrap test.

We will calculate 95% CIs for sensitivity, specificity, PPV, and NPV as bootstrap CIs.

#### Data Exclusion

Patients and their RAT results will be excluded from further analysis if no oropharyngeal swab has been collected for RT-PCR or if the sample has not been sent for reference RT-PCR testing at the DTU but has only been locally analyzed at the DCM.

### Ethical Approval

The study was evaluated by the National Committee on Health Research Ethics in the Danish Capital Region to be a method validation study without the need for approval by the committee (decision H-20068579). Access to test results for research was granted by the Capital Region of Denmark – Research and Innovation (R-20083753), and contact with participants without prior consent from them was granted by the board of directors at the hospitals at which the participating DCMs are situated.

## Results

### Summary

Results will be divided into several papers with different aims.

The first paper will report both retrospective and prospective performance data for RATs, with RT-PCR results as the reference method. The analytical performance data will be reported for 32 RATs and 1 SIBA-RT-PCR test, together with performance data from 43 RATs and 2 SIBA-RT-PCR tests from prospectively collected samples. Results will be reported overall and stratified into different C_q_ range groups.

The second paper will report results for RATs based on anatomical testing locations, comparing results from individuals who will be tested at multiple anatomical testing sites with the same RAT. Comparison of anatomical testing sites will be performed for 7 tests between the deep oropharyngeal cavity and the anterior nasal cavity, 3 tests between the deep oropharyngeal cavity and saliva, 2 tests between the anterior nasal cavity and saliva, and 2 tests between all 3 anatomical testing sites.

In the third paper, different anatomical testing sites will be compared for RT-PCR testing. Data from 2 different RT-PCR methods will be reported for RT-PCR from either samples collected in NEST buffer or samples collected as dry swabs. Approximately 600 SARS-CoV-2-positive and 600 SARS-CoV-2-negative individuals will be included for RT-PCR of liquid samples as deep oropharyngeal cavity swabs, anterior nasal cavity swabs, and saliva. Approximately for one-third of the individuals, deep nasopharyngeal cavity swabs will be included to allow comparison between all 4 anatomical sampling sites. In addition, approximately 400 SARS-CoV-2-positive and 100-SARS-CoV-2 negative individuals will be tested by dry deep oropharyngeal cavity swabs, anterior nasal cavity swabs, and saliva.

The fourth paper will focus on RATs that rely on central laboratory testing. Tests from 4 different manufacturers will be compared for analytical performance data on retrospective samples collected as deep oropharyngeal swabs. In addition, approximately 200 SARS-CoV-2-positive and 200 SARS-CoV-negative prospective samples will be compared for performance data from either deep oropharyngeal cavity swabs or anterior nasal cavity swabs as 1 test from either of the testing sites and 2 tests from both testing sites. In this way, 2 samples from each individual will be used for all 6 tests. Results will be compared to the results from RATs in the first paper.

In the fifth paper, 4 RATs will be tested both as professional use and as self-testing. The results from these 4 test comparisons will be reported, together with prospective performance data from 3 additional tests that will be collected as self-tests in the study.

The final paper will report the results from 2 breath tests performed in the study. Both tests will be tested on approximately 400 SARS-CoV-2-positive individuals and 200 SARS-CoV-2-negative individuals in the prospective part of the study. For 1 of the tests, the time to a positive test will be recorded and can be compared to results for the same test from deep oropharyngeal cavity swabs and anterior nasal cavity swabs.

## Discussion

### Summary

To the best of our knowledge, this is the largest study, both regarding the number of included tests and the number of included individuals, comparing RATs for SARS-CoV-2 on prospective samples that has been conducted so far. We will not only compare a large number of RATs but will also be able to address whether RATs as lateral-flow tests differ significantly from central laboratory–based RATs. Likewise, the anatomical test locations for both RATs and RT-PCR will be compared for multiple RATs, thereby adding information about testing sites for RATs and RT-PCR. Finally, the study will address self-testing versus professional testing and the use of expired air for RATs.

### Limitations

The study has 4 main limitations. First, as the RATs performed on participating outpatients in the prospective part of the study will be visually evaluated at the time of the test by the individual performing the test, and as this individual will be aware that the patient currently being tested by RATs has previously, within the last few days, tested positive by RT-PCR, the individual performing the test and visually evaluating the test result will be biased toward a positive result. Censoring the initial on-site visual evaluation by later evaluation of a picture will only be implemented if the initial on-site evaluation is negative and the subsequent validation by inspection of the picture by a test coordinator is positive. Initial on-site positive evaluations will not be subsequently censored by the test coordinator, as the outpatient testing team will get the opportunity to see the test strip from multiple angels, whereas the test coordinator is limited by review of the picture documentation. Thus, it is estimated that the sensitivity values obtained in the prospective part of the study will be biased toward higher sensitivity.

Second, as this study will be performed within a specific time frame at specific geographic locations in Denmark, the SARS-CoV-2 variants included in this study will reflect the variants present at that time and place and will likely not include all known variants. There are emerging data suggesting that different RATs will perform differently against different variants [18]; thus, the sensitivities for the different included RATs reported in this study may not reflect the actual sensitivity levels in future clinical test settings.

Third, the study will be performed in an unvaccinated population and vaccination toward SARS-CoV-2 may alter the sensitivity of RATs. Indeed, it has been shown that the peak virus load may be unaffected by vaccination, but vaccination can accelerate viral clearance, thereby narrowing the period for a positive RAT [19].

Finally, the study is designed to describe differences in sensitivity, both between the different RATs included and between different anatomical test sites. Although specificity values will be reported, the study is not powered to detect differences in specificity, neither between the different RATs included nor between different anatomical test sites.

### Conclusion

The study will compare the sensitivities of a large number of RATs for SARS-CoV-2 with those of RT-PCR and will address whether lateral flow–based RATs differ significantly from laboratory-based RATs. The anatomical test locations for both RATs and RT-PCR will also be compared.

## Abbreviations

BBQ: BlackBerry Quencher
BHQ1: Black Hole Quencher 1
Cq: quantification cycle
Cy5: cyanine fluorophore
DCM: department of clinical microbiology
DTU: Technical University of Denmark
FAM: 6-carboxyfluorescein fluorophore
HEX: hexachloro-fluorescein fluorophore
IFU: instructions for use
LC610: LightCycler Red 610 fluorophore
LDT: laboratory-developed test
NAAT: nucleic acid amplification technology
PCR: polymerase chain reaction
PoC: point of care
RAT: rapid antigen test
RT-PCR: Reverse Transcription Polymerase Chain Reaction
SIBA: strand invasion–based amplification
TCDK: TestCenter Denmark
UTM: Universal Transport Medium

## References

[1] Leuvering JHW, Thal PJ, van der Waart M, Schuurs AHWM (1980). Sol particle immunoassay (SPIA). J Immunoassay.

[2] Kozel TR, Burnham-Marusich AR (2017). Point-of-care testing for infectious diseases: past, present, and future. J Clin Microbiol.

[3] Moges B, Amare B, Belyhun Y, Tekeste Z, Gizachew M, Workineh M, Gebrehiwot A, Woldeyohannes D, Mulu A, Kassu A (2012). Comparison of CareStart™ HRP2/pLDH COMBO rapid malaria test with light microscopy in north-west Ethiopia. Malar J.

[4] Chou M, Kim S, Khim N, Chy S, Sum S, Dourng D, Canier L, Nguon C, Ménard D (2012). Performance of "VIKIA Malaria Ag Pf/Pan" (IMACCESS®), a new malaria rapid diagnostic test for detection of symptomatic malaria infections. Malar J.

[5] Lean WL, Arnup S, Danchin M, Steer AC (2014). Rapid diagnostic tests for group A streptococcal pharyngitis: a meta-analysis. Pediatrics.

[6] Bruning AHL, Leeflang MMG, Vos JMBW, Spijker R, de Jong MD, Wolthers KC, Pajkrt D (2017). Rapid tests for influenza, respiratory syncytial virus, and other respiratory viruses: a systematic review and meta-analysis. Clin Infect Dis.

[7] Merckx J, Wali R, Schiller I, Caya C, Gore GC, Chartrand C, Dendukuri N, Papenburg J (2017). Diagnostic accuracy of novel and traditional rapid tests for influenza infection compared with reverse transcriptase polymerase chain reaction. Ann Intern Med.

[8] Chartrand C, Leeflang MM, Minion J, Brewer T, Pai M (2012). Accuracy of rapid influenza diagnostic tests: a meta-analysis. Ann Intern Med.

[9] Rimoin AW, Walker CLF, Hamza HS, Elminawi N, Ghafar HA, Vince A, da Cunha AL, Qazi S, Gardovska D, Steinhoff MC (2010). The utility of rapid antigen detection testing for the diagnosis of streptococcal pharyngitis in low-resource settings. Int J Infect Dis.

[10] Hart A, Buck L, Morgan S, Saverio S, McLaughlin J (1997). A comparison of the BioStar Strep A OIA™ rapid antigen assay, group A selective strep agar (ssA™), and Todd-Hewitt broth cultures for the detection of group A streptococcus in an outpatient family practice setting. Diagn Microbiol Infect Dis.

[11] Ezike EN, Rongkavilit C, Fairfax MR, Thomas RL, Asmar BI (2005). Effect of using 2 throat swabs vs 1 throat swab on detection of group A streptococcus by a rapid antigen detection test. Arch Pediatr Adolesc Med.

[12] Allan-Blitz L, Klausner JD (2021). A real-world comparison of SARS-CoV-2 rapid antigen testing versus PCR testing in Florida. J Clin Microbiol.

[13] Wagenhäuser I, Knies K, Rauschenberger V, Eisenmann M, McDonogh M, Petri N, Andres O, Flemming S, Gawlik M, Papsdorf M, Taurines R, Böhm H, Forster J, Weismann D, Weißbrich B, Dölken L, Liese J, Kurzai O, Vogel U, Krone M (2021). Clinical performance evaluation of SARS-CoV-2 rapid antigen testing in point of care usage in comparison to RT-qPCR. EBioMedicine.

[14] Mistry DA, Wang JY, Moeser M, Starkey T, Lee LYW (2021). A systematic review of the sensitivity and specificity of lateral flow devices in the detection of SARS-CoV-2. BMC Infect Dis.

[15] European Commission EU Health Preparedness: A Common List of COVID-19 Rapid Antigen Tests and a Common Standardised Set of Data to Be Included in COVID-19 Test Result Certificates.

[16] Corman VM, Landt O, Kaiser M, Molenkamp R, Meijer A, Chu DKw, Bleicker T, Brünink S, Schneider J, Schmidt ML, Mulders DGjc, Haagmans BL, van der Veer B, van den Brink S, Wijsman L, Goderski G, Romette J-L, Ellis J, Zambon M, Peiris M, Goossens H, Reusken C, Koopmans MPg, Drosten C (2020). Detection of 2019 novel coronavirus (2019-nCoV) by real-time RT-PCR. Euro Surveill.

[17] Li D, Zhang J, Li J (2020). Primer design for quantitative real-time PCR for the emerging coronavirus SARS-CoV-2. Theranostics.

[18] Bekliz M, Adea K, Essaidi-Laziosi M, Sacks JA, Escadafal C, Kaiser L, Eckerle I (2021). SARS-CoV-2 rapid diagnostic tests for emerging variants. Lancet Microbe.

[19] Singanayagam A, Hakki S, Dunning J, Madon KJ, Crone MA, Koycheva A, Derqui-Fernandez N, Barnett JL, Whitfield MG, Varro R, Charlett A, Kundu R, Fenn J, Cutajar J, Quinn V, Conibear E, Barclay W, Freemont PS, Taylor GP, Ahmad S, Zambon M, Ferguson NM, Lalvani A, Badhan A, Dustan S, Tejpal C, Ketkar AV, Narean JS, Hammett S, McDermott E, Pillay T, Houston H, Luca C, Samuel J, Bremang S, Evetts S, Poh J, Anderson C, Jackson D, Miah S, Ellis J, Lackenby A (2022). Community transmission and viral load kinetics of the SARS-CoV-2 delta (B.1.617.2) variant in vaccinated and unvaccinated individuals in the UK: a prospective, longitudinal, cohort study. Lancet Infect Dis.

